# Effect of jaw opening on the stress pattern in a normal human articular disc: finite element analysis based on MRI images

**DOI:** 10.1186/1746-160X-10-24

**Published:** 2014-06-19

**Authors:** Qihong Li, Shuang Ren, Cheng Ge, Haiyan Sun, Hong Lu, Yinzhong Duan, Qiguo Rong

**Affiliations:** 1 Department of Orthodontics, School of Stomatology, Fourth Military Medical University, Xi’an 710032, China; 2 Department of Mechanics and Engineering Science, College of Engineering, Peking University, Beijing 100871, China; 3 Department of Stomatology, Afiliated Hospital of Academy of Military Medical Sciences, Beijing 100071, China

**Keywords:** Temporomandibular joint disc, Finite element analysis, Stress trajectory, Jaw opening

## Abstract

**Introduction:**

Excessive compressive and shear stresses are likely related to condylar resorption and disc perforation. Few studies have reported the disc displacement and deformation during jaw opening. The aim of this study was to analyze stress distribution in a normal articular disc during the jaw opening movement.

**Methods:**

Bilateral MRI images were obtained from the temporomandibular joint of a healthy subject for the jaw opening displacement from 6 to 24 mm with 1 mm increments. The disc contour for the jaw opening at 6 mm was defined as the reference state, and was used to establish a two dimensional finite element model of the disc. The contours of the disc at other degrees of jaw opening were used as the displacement loading. Hyperelastic material models were applied to the anterior, intermediate and posterior parts of the disc. Stress and strain trajectories were calculated to characterize the stress/strain patterns in the disc.

**Results:**

Both the maximum and minimum principal stresses were negative in the intermediate zone, therefore, the intermediate zone withstood mainly compressive stress. On the contrary, the maximum and minimum principal stresses were most positive in the anterior and posterior zones, which meant that the anterior and posterior bands suffered higher tensile stresses. The different patterns of stress trajectories between the intermediate zone and the anterior and posterior bands might be attributed to the effect of fiber orientation. The compression of the intermediate zone and stretching of the anterior and posterior bands caused high shear deformation in the transition region, especially at the disc surfaces.

**Conclusions:**

The stress and strain remained at a reasonable level during jaw opening, indicating that the disc experiences no injury during functional opening movements in a healthy temporomandibular joint.

## Introduction

The temporomandibular joint (TMJ), a load-bearing organ in the human body, contains an articular disc located between the glenoid fossa and the condyle that, during mandibular movements, plays an important role as a stress absorber during mouth function, resulting in stress reduction and redistribution within the joint [1].

The group of ‘temporomandibular disorders’ (TMD) comprises a number of related clinical problems involving pain and dysfunction of the masticatory system, the temporomandibular joint and its associated structures. The main cause of TMD has not yet been established [2], although functional overloading is considered to be a major etiological factor [1]. Indeed, excessive compressive and shear stresses are likely to be common sources of condylar resorption and disc perforation [3].

Stress distribution in the TMJ is hard to measure experimentally, and is thus poorly understood. However, finite element (FE) analysis is a promising research tool for evaluating dental biomechanics [4]. It can be used to analyze stress distribution patterns in the TMJ tissues after application of force or deformation. Two-dimensional (2D) and three-dimensional (3D) FE models have been used to simulate the in vivo biomechanics of the human TMJ [5-10]. Most previous studies focused on clenching behaviors, since maximum TMJ loading occurs during forceful clenching or aggressive episodes [11]. However, the TMJ is also sub-maximally loaded during many other activities, such as drinking, screaming, biting, and masticatory opening and closing [12]. The condylar movement during these various mandibular movements, especially jaw opening, produces remarkable ranges of disc mobility.

Until now, very few studies have reported dynamic simulation of the disc to explore disc displacement and deformation during jaw opening [12,13]. Biomechanical analysis of musculoskeletal system dynamics has been widely performed by applying rigid-body dynamics [14,15]. The distribution of forces in irregularly-shaped joint structures, however, cannot be analyzed, and deformations of the articular disc cannot be taken into account. Recently, methods combining 3D imaging and motion-tracking data (both optoelectric and electromagnetic) were introduced to study temporomandibular joint (TMJ) kinematics [16]. The combination of 3D TMJ anatomies and jaw tracking with six degrees of freedom permits a subject-specific dynamic analysis of TMJ loading during opening, closing and chewing. The location of the minimum intra-articular space was thought to bear the greatest force, although the nature of the articular disc gives it uneven thickness and irregular patterns of deformation, meaning that this relationship between force transfer and minimum intra-articular distance is somewhat ambiguous [12].

The aim of this study was to analyze the strain/stress pattern in the articular disc during jaw opening [1,6,9,10,17]. The contours of the disc at different opening distances were used as the displacement loading for FE analysis. Since this study was mainly a methodological validation of the applicability of the displacement loading based on MRI data, a 2D finite element model of the human mandible disc was applied.

## Materials and methods

### Magnetic resonance imaging and model reconstruction

**Figure 1:**
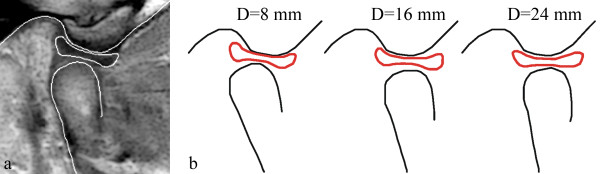
**Boundary tracing of the TMJ. (a)** MRI image of the TMJ at 6 mm of jaw opening. The contours of the fossa, disc and condyle are demarcated with white lines. **(b)** Diagrammatic representation of the contours of the fossa, disc and condyle at jaw opening of 8, 16 and 24 mm.

Bilateral MRI images were obtained from the TMJ of a 14-year-old female volunteer with no history of TMD. The MRI machine used was a high intensity 1.5-T magnet (Signa, Excite, General Electric, Chalfont St. Giles, United Kingdom) and a 3-in dual surface coil. An optimized proton density weighed fast spin echo sequence (repetition time 1600 ms/echo time 13.39 ms) was used for scanning. A field of view (FOV) of 100 × 150 mm and a slice thickness of 2 mm were used. Informed consent was obtained from the volunteer’s parents to participate in the study. We promise to protect life, health, dignity, integrity, right to self-determination, privacy, and confidentiality of personal information of research subject. Ethical permission has been offered by Scientific research and clinical application of medical technology ethics committee of Afiliated Hospital of Academy of Military Medical Sciences with a reference number of KY-2013-03-06. MRI was taken in the sagittal direction, perpendicular to the long axis of the condyle. A mouth gag was placed in the mouth before image acquisition, and the subject was asked to close her mandible onto the mouth gag. After one cycle of imaging, she was asked to press a button on the gag to increase jaw opening by 1 mm. Contiguous 2 mm-thick sagittal slices of the dentition were obtained at each opening distance. Sagittal MRI images of the same slice were selected for 19 different opening distances at 1 mm increments. The minimum opening distance between central incisors is 6 mm by the mouth gag. The range of jaw opening was from 6–24 mm. The contours of the articular fossa, articular disc and condyle were traced by a trained dentist (Figure 1).

To make sure that slices from the same position, the volunteer’s head was fixed in the same place. All MR images were taken by the same slice. Besides, the images were registered in Matlab (version: 2010a, MathWorks, USA) by alignment of the articular fossa which is considered no movement during jaw opening.

### Finite element modeling and analysis

**Figure 2:**
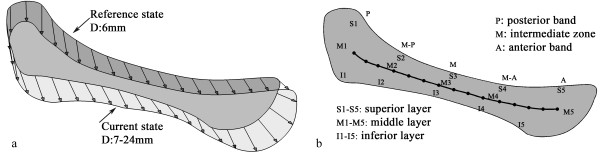
**Loading conditions and zoning of the disc. (a)** Loading conditions of the disc, obtained by the displacement of corresponding nodes on the boundary of disc in the reference state (6 mm opening; grey outline) and the disc in the current states at varying distances of opening (7–24 mm; white outline). **(b)** Diagram showing the fifteen zones analyzed in this study, comprising posterior (P), medial-posterior (M-P), medial (M), medial-anterior (M-A) and anterior (A) positions in each of the superior (S), middle (M) and inferior (I) layers. The central line represents the path used to evaluate the stretch of the disc.

The contour of the disc with the jaw open at 6 mm was defined as the reference state, and was used to establish a two dimensional finite element model of the disc. The initial stress state of the FE model was assumed to be zero. All other disc contours were meshed in the same way as for the FE model. For a given opening distance, the difference in the coordinates of the corresponding nodes (i.e. the displacement of the boundary nodes of the FE model) was used as the displacement loading, as shown in Figure 2a. Altogether, 18 simulations (jaw opening range: 7–24 mm) were performed. The stress analysis was executed by the FE software, ANSYS 14.0 from ANSYS Inc. (Houston, USA).

The TMJ disc is a dense fibrocartilaginous structure [1] and its mechanical behavior has been proven to be nonlinear, anisotropic and time-dependent [18-20]. In addition, there are differences in the mechanical behavior of the anterior, posterior and medial parts of the disc [10]. In recent studies, Perez [6] developed an accurate TMJ model using a fiber-reinforced porohyperelastic material for the articular disc. However, the constants for this material model were obtained from dogs. In the current study, we also used a hyperelastic model of the TMJ disc, described by the experimental response function. For hyperelastic materials, the stress–strain relationship derives from a strain energy density function:

$$S_{\mathrm{ij}}=\frac{\partial W}{\partial E_{\mathrm{ij}}}$$

where S_ij_ is a component of the second Piola-Kirchhoff stress tensor; W is the strain energy density: and E_ij_ is a component of the Lagrangian strain tensor.

The Cauchy stress components of a volumetrically constrained material can be shown to be [21]:

$$\sigma_{\mathrm{ij}}=-p\delta_{\mathrm{ij}}+\mathrm{dev}\left[2\frac{\partial W}{\partial I_{1}}b_{\mathrm{ij}}-2I_{\alpha }\frac{\partial W}{\partial I_{2}}b_{\mathrm{ij}}^{-1}\right]$$

where δ_ij_ is the Kronecker delta; p is the pressure; and b_ij_ is the left Cauchy-Green deformation tensor. The deviatoric stress is therefore determined solely by the deformation and the response functions (derivatives $\frac{\partial W}{\partial I_{1}}$, $\frac{\partial W}{\partial I_{2}}$ and $\frac{\partial W}{\partial J}$), which are determined analytically for the hyperelastic potentials in isotropic and anisotropic hyperelasticity.

The parameters of the response functions (shown in Table 1) were obtained from the experimental data reported by Kang [18], where Kang and coworkers performed tensile tests of the anterior, intermediate and posterior bands along the medial-lateral axis of 13 human TMJ discs. Considering the fiber directions in the disc, the mechanical properties of the anterior and posterior bands can be characterized with data obtained from the intermediate zone, since the fibers of the intermediate zone run perpendicular to the axis of the tensile test [10]. Conversely, the elastic constants related to the intermediate zone can be obtained from tests conducted on tissue in the anterior band. Since tests performed on the posterior band were along the fibers, it was expected to be stiffer than the intermediate zone; however, it turned out to be softer. This suggests that the test may have been conducted too close to the end of the disc, so we eliminated the posterior band data. Since no compression test data can be found in literatures, the disc was assumed to have the same material behavior in compression and tension.To evaluate stress variation in the anterior and posterior bands and the intermediate zone, a path was defined in the articular disc. In addition, the disc was divided into 15 zones to characterize the stress/strain patterns during jaw opening (Figure 2b).

**Table 1:** Material data of the anterior, intermediate and posterior parts of the disc

**Strain**	**Stress (MPa) AB/ PB**	**Stress (MPa) IZ**
0	0	0,0
0.01	0.0233	0.0311
0.02	0.0506	0.0638
0.03	0.0801	0.0988
0.04	0.1268	0.1524
0.05	0.1999	0.2186
0.06	0.2862	0.3267
0.08	0.6143	0.6628
0.1	0.9284	0.9815
0.13	1.1316	1.351
0.15	1.2979	1.6351
0.17	1.388	1.7575
0.2	1.4688	1.8083

AB: anterior band; PB: posterior band; IZ: intermediate zone.

## Results

**Figure 3:**
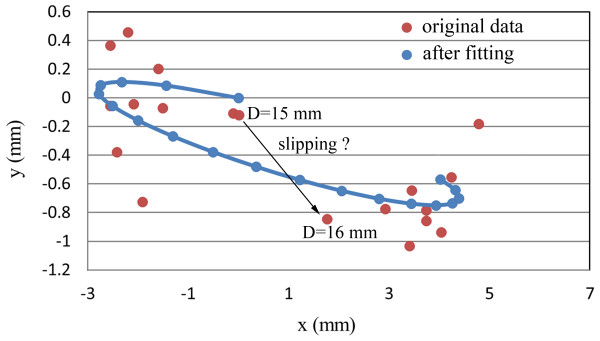
Trajectory of the disc center during jaw opening.

**Figure 4:**
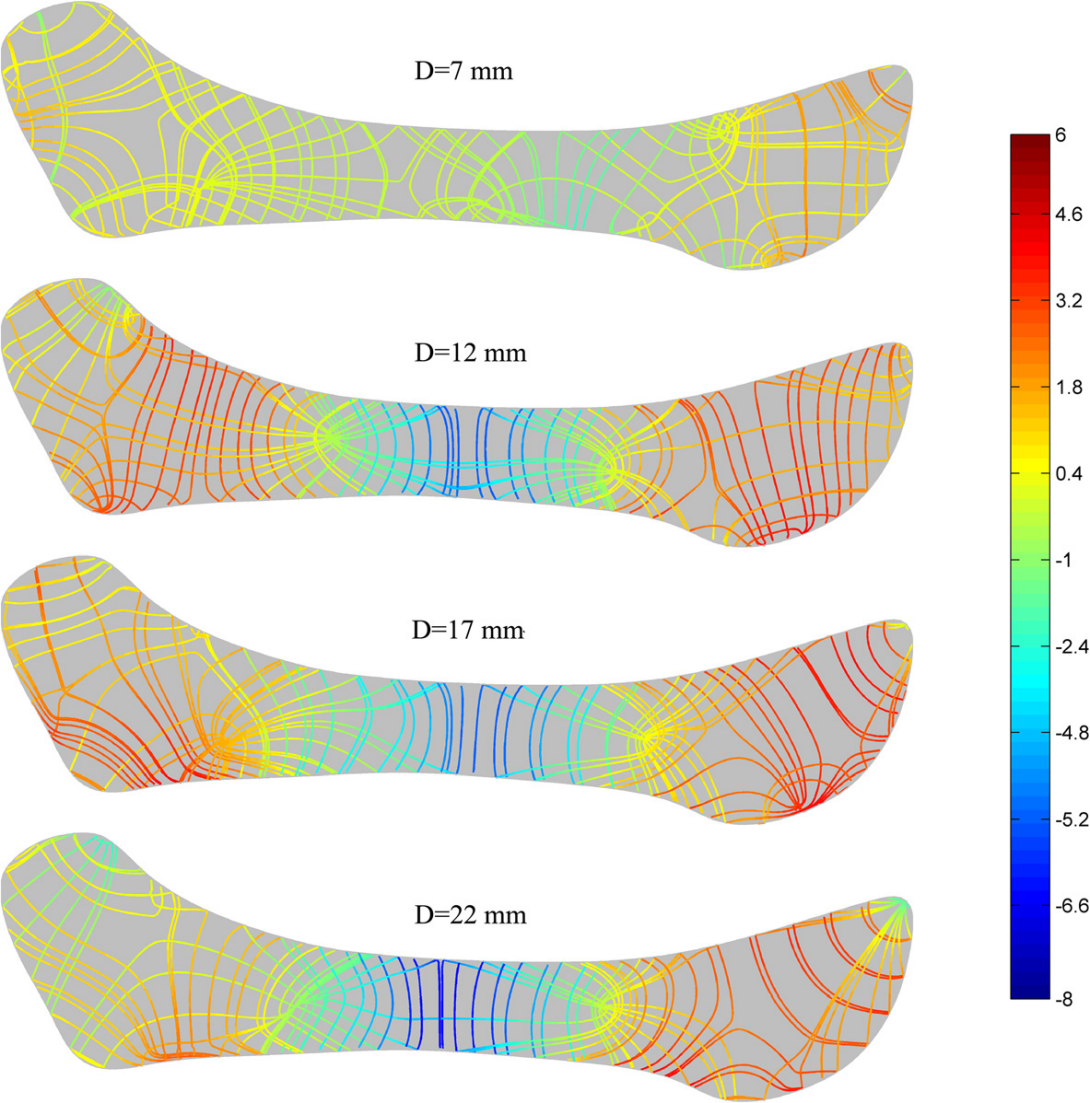
Stress trajectories of the disc at jaw opening of 7, 12, 17 and 22 mm (MPa).

**Figure 5:**
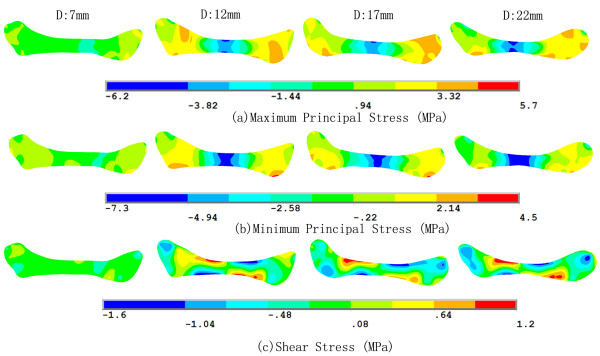
Stress distribution of the disc at jaw opening of 7, 12, 17 and 22 mm (MPa).

**Figure 6:**
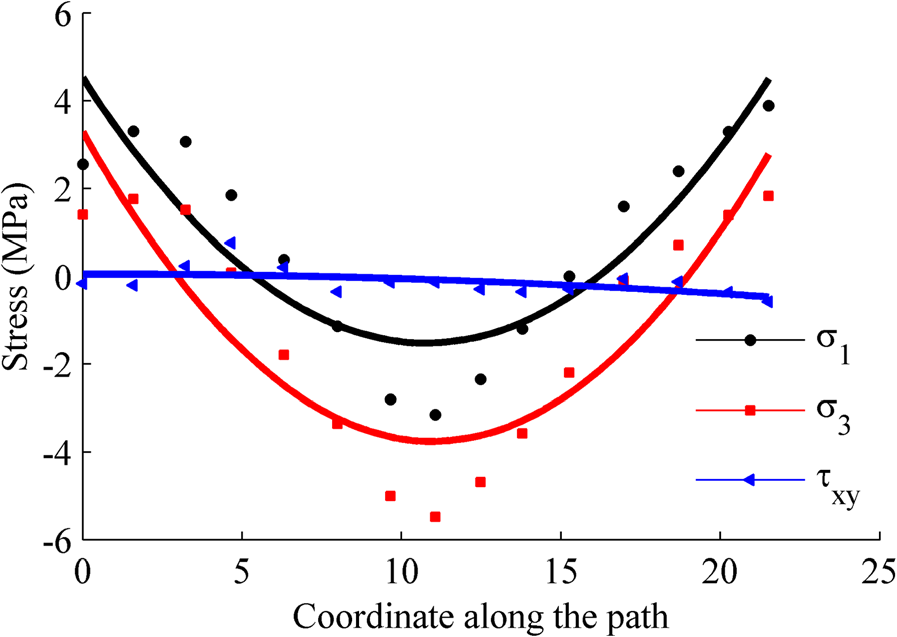
**Stress along the postero-anterior path on the disc at 18 mm of jaw opening.** σ_1_: the first principal stress, σ_3_: the third principal stress, τ_xy_: the shear stress.

Figure 3 shows the positions of the disc center during the jaw opening process. These appeared to be distributed randomly and the movement of the disc center was not strongly correlated with the opening distance. After fitting, the trajectory of the disc center was ovoid in shape. This result shows that the opening distance does not describe the disc position on its own. Although jaw opening is a continuous movement, the disc center might undergo intermittent and rapid slippage or displacement. Indeed, the disc center jumped suddenly when the jaw opened from 15 mm to 16 mm, perhaps as a consequence of friction between the disc and the condyle.Figure 4 shows the stress trajectories of the disc at jaw opening of 7, 12, 17 and 22 mm, and the corresponding stress distributions are shown in Figure 5. Except at 7 mm, where the stress level was relatively low, the stress trajectories had similar patterns in each condition. It can be observed that both the maximum and minimum principal stresses were negative in the intermediate zone. Stress trajectories run vertically and horizontally, with the disc bearing mainly the vertical pressure. The maximum and minimum principal stresses were most positive in the anterior and posterior bands. The tensile stresses in the anterior band may be caused by stretching of the disc by the lateral pterygoid muscle, while that in the posterior band may be caused by contracting of the bilaminar zone. The transition from compressive stress in the intermediate zone to tensile stress in the anterior and posterior bands did not occur smoothly, instead being accomplished at a singular point in the transition area.Figure 6 shows the pattern of stresses on the disc along the posterior-anterior path at jaw opening of 18 mm. The fitted curves of the maximum and minimum principal stresses appeared to be funnel-shaped, confirming that the posterior and anterior bands were stretched, whereas the intermediate zone bore compressive stress. The shear stress was relatively small when compared with the principal stresses, although were appreciable in the whole disc.

**Figure 7:**
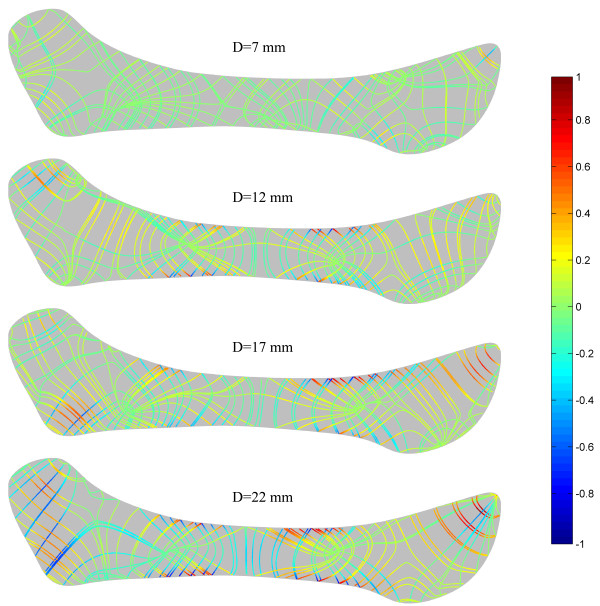
Strain trajectories of the disc at jaw opening of 7, 12, 17 and 22 mm.

**Figure 8:**
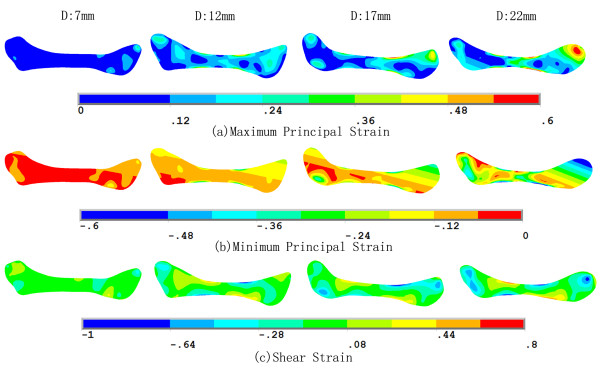
Strain distribution of the disc at jaw opening of 7, 12, 17 and 22 mm.

**Figure 9:**
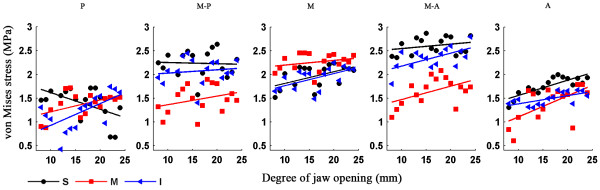
**Relationship of von Mises stress and the opening distance.** Von Mises stress in the superior (S), middle (M) and inferior (I) disc layers is plotted against the opening distance.

**Figure 10:**
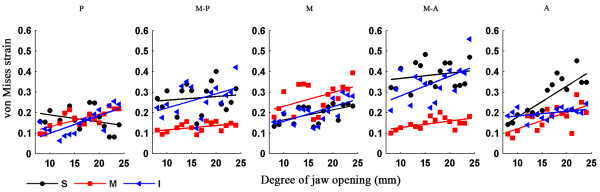
**Relationship of von Mises strain and the opening distance.** Von Mises strain in the superior (S), middle (M) and inferior (I) disc layers is plotted against the opening distance.

Figure 7 shows the strain trajectories of the disc at jaw opening of 7, 12, 17 and 22 mm, the patterns of which were similar to those of the stress trajectories. The corresponding strain distributions are shown in Figure 8. The shear deformation can be observed more clearly here. The maximum shear strain at any point is half the difference between the two principal strains; thus the difference in the two trajectories is proportional to the maximum shear strain. A sharp contrast between the two trajectories corresponds to a larger shear strain. It can be easily found that the shear strain concentration occurred at the interfaces between the intermediate zone and the anterior and posterior bands. The intermediate zone maintained contact with the condyle and the temporal bone during jaw opening, so its superior and inferior surfaces were compressed vertically. Therefore, the disc was precluded from relative slippage by the actions of friction forces. Besides, retrodiscal tissue may also prevent the disc from slipping [6,21]. The compression of the intermediate zone and stretching of the anterior and posterior bands caused high shear deformation in the transition region, especially at the disc surfaces.Figure 9 shows that, in almost all regions of the disc, the von Mises stress increased with the opening distance. The only exception is the superior layer of the posterior band, where the von Mises stress decreased as the jaw opened. The highest stress occurred in the superior medial-anterior region. In general, the stress increased very gently, indicating that jaw opening is unlikely to lead to any significant risk of stress damage. Similar results are shown in Figure 10, which shows the relationship between von Mises strain and the opening distance.

## Discussion

The TMJ is perhaps the most heavily used load-bearing joint in the human body, so its biomechanical balance has great significance to its function [21]. The articular disc in the TMJ acts as a stress cushion during TMJ activity, so analysis of stress distribution throughout this disc during function is of great importance.

The opening movement is highly relevant to the response of articular discs to this motion [10], but the position of the disc cannot be determined only by the opening distance; other influential parameters are required to describe the configuration of this disc. In previous studies, FE models of the TMJ were developed to investigate stress and reaction forces within the TMJ during jaw opening [1,6,17,22,23], closure [17,24,25], clenching [21,26-28], and chewing [29] with active masticatory muscle forces [11,17,29] the favored method of load application. To avoid the experimentally difficult estimation of muscle forces [24], alternative loading conditions such as displacement of the condyle during clenching [10] and jaw opening [1] have been used. However, few studies have reported simulation of disc deformation during jaw opening that uses the disc contours as the displacement loading. In the present study, we observed displacement of the disc boundary by measuring the contours of the disc in MRI images.

The mechanical behavior of the TMJ disc, when investigated experimentally in humans [18], dogs [19] and pigs [20], was found to be nonlinear, anisotropic and time-dependent, and varied between different regions of the disc. However, in most previous studies [1,5,6,10,23,24], the material properties of the disc were considered as entirely homogeneous. Recently, Perez [6] developed an accurate TMJ model that used a fiber-reinforced porohyperelastic constitutive model for the disc, where the constants for material models were extrapolated from tensile tests in dogs [19].

Similarly, an experimental response function was applied in this study to describe the hyperelastic property of the disc. Parameters of the response function were obtained from tensile tests of human TMJ discs [15]. The incompressibility of the disc was not considered in this study, since the jaw opening here was a quasistatic process, the water seepage in the disc was therefore neglected. The stress/strain trajectory patterns show that the fiber orientation has a significant influence on disc deformation, meaning that it is reasonable to consider the effects of fiber orientation and distribution in the disc. Because of its hyperelasticity, the disc endures high strains and relatively low stresses. During jaw opening, the intermediate zone bears mainly compressive stress, in agreement with previous studies [6]. Conversely, the anterior and posterior bands bear mainly tensile stress. Other studies [30,31] have found the same situation during clenching. These results indicate that the function of the disc is companied by combined impact of stretching of the ligaments and compression of the condyle and the articular fossa. The von Mises stress and strain increase gradually with the opening distance, but remain at a reasonable level, indicating that the opening movement does no harm to the disc, consistent with expectations for the performance of a healthy disc. Likewise, Tanaka [22] also found that the von Mises stress increased in the disc as jaw opening progressed. The only exception in the present study is the superior layer of the posterior band. This may be caused by a big clockwise rotation of the disc, and therefore reduce the stress in that zone.

Some studies have reported that perforation of the disc may arise due to high shear stresses [32-34]. Our simulation results show that the highest shear stress occurs at the boundaries of the middle-anterior and middle-posterior regions. This may be due to the transition of the loading conditions from compressive in the intermediate zone to tensile in the anterior and posterior bands.

There are some limitations in the present study. Only oblique sagittal jaw displacement has been considered using this 2D FE model, so our results are not as vivid as they might have been with a 3D model. Also, some simplifications were made with respect to the displacement loading: displacement of the disc boundary was obtained from the corresponding node pairs of the two disc configurations, which may lead to stress concentration on parts of the boundary. However, most simulation results are highly reasonable.

The present work represents an innovative trial of a more accurate technique for predicting the stress response during similar motion problems, not only for discs but also for other organs, tissues and joints in the human body.

## Conclusion

The simulations showed that the highest compressive stress occurred in the intermediate zone, whereas the anterior and posterior bands experienced mainly tensile stress. Fiber orientation had a significant effect on the stress/strain patterns. The stress and strain increased slightly with the opening distance, but were remarkably stable. The highest shear stress was at the interfaces between the medial-anterior and medial-posterior zones. Generally, the stress and strain remained at a reasonable level during jaw opening, indicating that the disc experiences no injury during functional opening movements in a healthy joint.

## Competing interests

There are no potential competing interest to disclose.

## Authors’ contributions

QR and YD initiated the investigation and designed the study. QL traced contours of the temporomandibular joint. SR preformed the finite element analysis. QL and SR drafted the manuscript. QR and YD critically reviewed and revised the manuscript. CG, HS, HL participated in MR image acquisition and analysis. All authors read and approved the final manuscript.
